# The High Five Model: Associations of the High Factors With Complete Mental Well-Being and Academic Adjustment in University Students

**DOI:** 10.5964/ejop.v15i4.1759

**Published:** 2019-12-19

**Authors:** Alejandro Castro Solano, Alejandro César Cosentino

**Affiliations:** a Consejo Nacional de Investigaciones Científicas y Técnicas (CONICET), Buenos Aires, Argentina; b Facultad de Psicología, Universidad de Buenos Aires, Buenos Aires, Argentina; c Departamento de Psicología, Facultad de Ciencias Sociales, Universidad de Palermo, Buenos Aires, Argentina; University of Wroclaw, Poland

**Keywords:** academic achievement, well-being, personality traits, High Five model, personality test

## Abstract

Traditionally, models of positive personality traits have referred to moral characteristics. The High Five Model (HFM) is a factor model of individual positive traits based on an inductive psycho-lexical approach. Unlike other models, in the HFM the positive characteristics were freely determined by lay people, beyond any moral tones. The HFM comprises the following factors: erudition, peace, cheerfulness, honesty, and tenacity, known as “the high factors.” This model was shown to positively exceed the capacity of normal personality to predict emotional, social, and psychological well-being. Additionally, this model is negatively associated with non-transmissible diseases, psychopathological symptoms, and psychopathological personality traits. This study aimed to increase the validation of the HFM, by analyzing the relationships among this model and positive mental health, psychopathological symptoms, academic adjustment, and academic performance in university students. Another objective was to study the association between complete mental well-being (i.e., high well-being and low psychopathological symptomatology) and the high factors of the HFM. The sample consisted of 256 university students. Correlations were calculated, and the two-step cluster analysis was used to obtain profiles. The results showed that tenacity and erudition high factors are positively associated with academic achievement and academic adjustment. Finally, each of the high factors was positively associated with complete mental well-being. The HFM has a broad scope, as it is related not only to psychological variables (e.g., well-being or psychopathological symptomatology) but also to academic performance (e.g., adjustment and achievement) in university students.

Theory-driven or data-driven studies on positive characteristics have focused on one type of positive traits, namely moral traits (e.g., virtues and character strengths), excluding abilities or non-moral features (e.g., Dahlsgaard, Peterson, & Seligman, 2005). From a theory-driven perspective, Peterson and Seligman (2004) proposed a classification into six virtues, developed from the qualitative analysis of philosophical and religious texts of Eastern and Western traditions. Afterward, a series of academic debates on these virtues led to the proposal of 24 strengths of character that hypothetically correspond to those six virtues. Although this classification of positive traits has a strong influence on Positive Psychology (Seligman & Csikszentmihalyi, 2014), many studies have failed to confirm it (e.g., McGrath, 2014; Noftle, Schnitker, & Robins, 2011). From a data-driven approach, different virtue structures or models have been proposed (e.g., Cawley, Martin, & Johnson, 2000; De Raad & van Oudenhoven, 2011; Morales-Vives, De Raad, & Vigil-Colet, 2014). A pioneering study on the topic was conducted by Walker and Pitts (1998), who asked young, middle-aged, and elderly adults of both genders to identify the attributes of a highly moral person and then requested some students to classify these moral descriptors into groups. Finally, the authors analyzed the data, using hierarchical cluster analysis and multidimensional scaling, and obtained six clusters of attributes of moral characteristics.

The High Five Model (HFM) was recently proposed for identifying individual positive traits from lay people’s perspective (Cosentino & Castro Solano, 2017). These traits, known as high factors, are erudition, peace, cheerfulness, honesty, and tenacity. The aim underlying the development of this model was to avoid constraining positive psychological characteristics to moral concepts and allow any human psychological characteristic to be considered if lay people perceived them as positive. The HFM was consistently developed using a psycho-lexical data-driven approach, adopting statistical and syntactic criteria, rather than a semantic selection criterion, in an effort to exclude any theoretical influence from an academic point of view.

To develop this model of socially shared positive individual characteristics, 745 participants (399 women, *M* = 37.7, *SD* = 15.1, range 18–90 years) generated a list of 854 elements that made up the initial corpus of words (Cosentino & Castro Solano, 2017). The corpus consisted of words that lay people used in their daily lives. No limits had been established on the type of positive features that could be mentioned, and therefore the corpus included moral characteristics (e.g., reliable) as well as characteristics related to abilities (e.g., intelligence) or lacking moral connotations (e.g., serenity). Syntactic and statistical procedures were used to reduce the corpus to a manageable list of positive psychological individual characteristics. As a result, 140 items were retained and included in an inventory. The model exploration sample consisted of 516 participants (286 women, *M* = 35.2, *SD* = 13.5, range 18–80). The participants were asked to indicate to what extent the items described them (scale ranging from 1 to 7). To determine the number of factors, an exploratory factor analysis was run, using parallel analysis based on minimum rank factor analysis. Additionally, the oblique Crawford-Ferguson (CF) Quartimax rotation was used to obtain a cleaner factor structure, which resulted in a set of 57 items, corresponding to five positive factors. The model refinement sample consisted of 484 participants (285 women, *M* = 35.1, *SD* = 14.0, range 18–79), who indicated to what extent the positive characteristics described them (range 1–7). A new factor analysis was conducted to reduce the number of items and obtain a factor model with at least four items, omega reliability ≥.80 per factor, and good fit to data. Thus, the High Five Inventory (HFI) was composed of 23 items distributed into five subscales of socially shared positive human characteristics (Table 1). The results, analyzed with a robust diagonally weighted least squares estimator, were CFI > 0.95, SRMR < 0.05, RMSEA < 0.06, and omega reliabilities were >.80 for each factor. Next, the analysis of the HFI scores obtained from a sample of 1,118 participants (564 women, *M* = 40.4, *SD* = 14.2, range 18–92) confirmed the good fit of the model to data using robust analyses (CFI > 0.96, SRMR < 0.05, and RMSEA < 0.07), as well as the good reliability of each subscale (omegas > .80). Not only did each high factor show positive associations with emotional, psychological, and social well-being from the hedonic and eudemonic traditions (Westerhof & Keyes, 2010), but the HFM also predicted each of the three dimensions of positive mental health, accounting for the variance above and beyond the amounts accounted for by the Big Five factors or facets. Another study consistently showed that high scores on the high factors are related to mental health indicators (according to the categorical diagnosis of flourishing/languishing; Keyes, 2005). Conversely, the high factors are negatively associated with disease indicators, such as the risk of medical non-transmissible diseases, psychopathological symptoms, and psychopathological personality traits (Castro Solano & Cosentino, 2017). The term "high" for the factors of the HFM was selected, on the one hand, to show that these factors are linked to the individual characteristics that lay people value highly or positively. On the other hand, they are considered positive poles, in relative terms, of the Big Five model factors. It was found that the high factors of the HFM are positively associated with the Big Five factors. The high factor erudition is positively associated with the Big Five personality factor openness, peace with emotional stability, cheerfulness with extraversion, honesty with agreeableness, and tenacity with conscientiousness (Cosentino & Castro Solano, 2017). However, these relations should not be taken as exclusive and biunivocal but, rather, each factor of the HFM is ***primarily*** linked to one of the factors of the Big Five model, but ***secondarily*** to other factors.

**Table 1:** High Factors, Definitions, and Items of the High Five Inventory (HFI)

High Factors	Description	HFI Items
Erudition	The positive trait of knowledge, expressed in thinking about solutions, creating things, desires to learn.	Intelligent, wise, visionary, cultured, genius, ingenious
Peace	The positive trait of balance, expressed in thinking about calm, believing that there is a solution to everything, or that things will happen in due course.	Patient, tolerant, tranquility, serenity
Cheerfulness	The positive trait of excitement, expressed in desires to make people laugh, have fun and help others to have fun, or to have amusing ideas.	Humor, pleasant, funny, amusing
Honesty	Moral positive trait, expressed in desires to show one´s true self, to tell the truth, or to be a good person.	Loyal, reliable, values, transparent, truthful
Tenacity	The positive trait of volition, expressed in thinking about goals, achieving goals, or believing that goals require effort.	Dedicated, persistent, effort, industrious

The results of previous studies on moral traits (e.g., virtues or character strengths) resemble the findings of the studies on the HFM, since moral traits are generally predictors of life satisfaction and different types of psychological well-being (e.g., Castro Solano & Cosentino, 2016; Cosentino & Castro Solano, 2015; Park, Peterson, & Seligman, 2004). In addition, moral traits are predictors of positive outcomes, such as good job performance (Harzer & Ruch, 2014) or good academic achievement (Cosentino & Castro Solano, 2012; Lounsbury, Fisher, Levy, & Welsh, 2009).

It is necessary to consider why personological variables, in this case positive traits, are expected to correlate with academic performance, given that the personality variables were not designed to predict such an outcome (Ackerman & Heggestad, 1997). A corollary of the lexical hypothesis is that the more valued a personological characteristic, the more descriptors for this characteristic will be found in the natural language. If the lexical hypothesis is correct, then the dimensions of the HFM should be related to behaviors and outcomes that have been independently recognized as important (Poropat, 2009). Performance in both the academic and labor fields is determined by the capacity to perform, the opportunity to perform, and the willingness to perform. Capacity incorporates knowledge, skills, and intelligence; opportunity to perform is affected by environmental constraints and resources, including socioeconomic resources (Traag, van der Valk, van der Velden, de Vries, & Wolbers, 2005), while willingness to perform reflects motivation, cultural norms, and personality (Blumberg & Pringle, 1982). Recent meta-analyses have provided evidence that both capacity and opportunity to perform are correlated with academic performance (Poropat, 2009). As regards willingness to perform, personality variables may contribute directly but have been indirectly linked through their associations with motivation (Judge & Ilies, 2002). Similarly, Chamorro-Premuzic and Furnham (2006) argued that correlations between academic performance and personality measures would mirror corresponding correlations of intelligence with personality. Therefore, not only do the personality variables predict important socially valued psychological outcomes, such as academic achievement and adjustment, but they are also directly related to the individual's willingness to perform.

A validation study of the HFM model was conducted among university students attending different undergraduate courses. The complete optimal functioning, the construct we related to the HFM, was assessed in terms of academic performance (academic adjustment and academic achievement), indicators of positive mental health (i.e., high hedonic and eudemonic well-being), and absence of psychopathological symptoms. It was assumed that the HFM makes it possible to predict positive results. Therefore, the objectives of this research were 1) to study the associations of the HFM factors with self-reported academic achievement and self-perceived academic adjustment in university students, and 2) to study the associations of the HFM factors with indicators of positive mental health and absence of psychopathological symptoms in university students.

## Method

### Participants

The sample consisted of 256 university students (125 men, 48.8%, and 131 women, 51.2%), with a mean age of 27.55 years (*SD* = 7.38, range 18–56), mostly residents from Buenos Aires city (*n* = 173, 67.6%) and Buenos Aires suburbs (known as “Greater Buenos Aires,” *n* = 60, 26.4%). A low percentage (*n* = 23, 9%) resided in other cities of Buenos Aires Province. Almost three-quarters of the participants were working and studying at the time of the study (*n* = 187, 73%), attending either state-run (*n* = 111, 43.4%) or private (*n* = 145, 56.6%) universities.

Half of the participants were single (*n* = 139, 54.3%), 22.7% (*n* = 58) were living with a partner or married, 21% were dating (*n* = 54), and the remaining 2% (*n* = 5) were divorced. Most participants reported having a middle (*n* = 173, 67.6%) or lower-middle (*n* = 30, 11.7%) socioeconomic status (SES), while 20% reported an upper-middle SES (*n* = 51, 11.7%).

This was a convenience sample of urban Argentinean university students who study and work to afford their education. Participation was voluntary, consented, and anonymous. No economic incentives were given for participation. The sample was recruited by advanced psychology students doing their research practice at the end of their undergraduate studies.

### Instruments

#### High Five Inventory

The High Five Inventory (HFI; Cosentino & Castro Solano, 2017) is an instrument to assess the HFM factors, known as high factors: erudition, peace, cheerfulness, honesty, and tenacity. This instrument was developed through an inductive procedure originating from lay people’s point of view about human positive characteristics (moral or non-moral). The scale consists of 23 items. Participants are asked to answer each item (e.g., *I am patient*) on a Likert-type scale ranging from 1 (*Never*) to 7 (*Always*). The higher the score of each HFI subscale, the higher the high factor. The HFI has convergent and divergent validity in relation to Peterson and Seligman’s (2004) Values ​​in Action classification. The HFI also has incremental validity, that is, beyond the factors or facets of the Big Five, the HFI increased the prediction of the three dimensions of Positive Mental Health model of Keyes (2005). Additionally, the HFI presented a good fit to the data from both the initial (e.g., CFI = 0.968) and the confirmation (e.g., CFI = 0.963) samples. The alpha and omega reliabilities for each factor were greater than .80. The internal consistency of the high factors in this sample is shown in Table 2.

**Table 2:** Descriptive Statistics of the High Factors, Psychopathological Symptoms, Well-being Dimensions, Academic Adjustment, and Academic Achievement

Variable	*M*	*SD*	Cronbach’s α
HFI honesty	5.98	0.94	.88
HFI tenacity	5.46	1.07	.86
HFI cheerfulness	4.87	1.20	.90
HFI erudition	4.50	1.08	.85
HFI peace	4.46	1.20	.85
SCL-27 psychopathological symptoms	0.66	0.53	.92
MHC emotional well-being	3.66	0.90	.81
MHC personal well-being	3.49	0.85	.79
MHC social well-being	2.16	1.06	.80
Academic adjustment	3.69	0.79	.78
Academic achievement	7.36	1.00	-

*Note*. HFI = High Five Inventory; MHC = Mental Health Continuum; *SD* = standard deviation.

#### Symptom Checklist 27

The Symptom Checklist 27 (SCL-27, Hardt & Gerbershagen, 2001) is a shorter version of the SCL-90-R (Derogatis, 1975), originally developed to assess patients with chronic pain. The SCL-27 is a short and multidimensional detection instrument for mental health problems and it consists of six scales of symptoms: (I) depressive symptoms, (II) dysthymic symptoms, (III) vegetative symptoms, (IV) agoraphobic symptoms, (V) symptoms of social phobia, and (VI) symptoms of mistrust. In addition, it has a Global Severity Index (GSI) of the person's current discomfort. Items are answered on a 5-point Likert scale ranging from 0 (*Not at all*) to 4 (*Extremely*). In this study, the GSI was used as an indicator of symptomatology. The SCL-27 dimensions were strongly positively associated with each other (Kuhl et al., 2010). The discrimination capacity between general and clinical population is similar to that of the SCL-90-R. This short version is often recommended for screening in clinical and research studies (Müller, Postert, Beyer, Furniss, & Achtergarde, 2010) because it has demonstrated adequate levels of sensitivity, specificity, discrimination, and internal consistency, compared with other short versions of the SCL-90-R. The alpha reliability of the general scale for the sample of this study was .92.

#### Mental Health Continuum—Short Form

The Mental Health Continuum—Short Form (MHC-SF) (Keyes, 2005) is composed of 14 items that evaluate emotional (3 items), psychological (6 items), and social (5 items) well-being. Participants answer how they have felt over the previous month, in a Likert-format option, ranging from 0 (*Never*) to 5 (*Every day*). The MHC-SF has shown good internal consistency (>.70) and adequate validities (3-factor structure: emotional, social, and psychological well-being) in studies conducted in adult populations in the United States, China, Italy, Iran, Canada, Poland, and South Africa (Gallagher, Lopez, & Preacher, 2009; Gilmour, 2014; Joshanloo, Wissing, Khumalo, & Lamers, 2013; Karaś, Cieciuch, & Keyes, 2005, 2014; Lamers, Westerhof, Bohlmeijer, ten Klooster, & Keyes, 2011; Petrillo, Capone, Caso, & Keyes, 2015; Robitschek & Keyes, 2009; Yin, He, & Fu, 2013). The Argentinean version of the MHC-SF was used in the present study. The psychometric properties of this version, such as adequate reliability, factor structure, and convergent validity, have been confirmed (Lupano Perugini, de la Iglesia, Castro Solano, & Keyes, 2017). In the sample of the present study, the internal consistency was α = .81 for emotional well-being, α = .79 for psychological well-being, and α = .80 for social well-being.

#### Academic Adjustment Scale

The Academic Adjustment Scale (Anderson, Guan, & Koc, 2016, adapted by Castro Solano & Cosentino, 2017) was designed to assess international students’ self-perception of adjustment to university life. The Argentinean version of this scale includes five out of the nine original items. This inventory is answered on a 5-Likert-type scale, ranging from 0 (*Almost never*) to 5 (*Always*). The items of the Argentinean adaptation are grouped into a single factor that denotes satisfaction with their adjustment. The internal consistency for this sample was high (omega = .81).

#### Academic Achievement

Participants’ self-reports of academic grades (average, range 0–10) were used. A meta-analysis has shown a strong correlation (*r* = .90) between self-reported academic grades and objective academic achievement as reported by universities (Kuncel, Credé, & Thomas, 2005). Therefore, self-reported academic achievement is widely used in contemporary research (e.g., Bücker, Nuraydin, Simonsmeier, Schneider, & Luhmann, 2018; Leyrer-Jackson & Wilson, 2018), as an adequate indicator of objective grades.

#### Sociodemographic Data

A survey was designed to gather sociodemographic characteristics of the population (e.g., sex, age, type of educational institution).

### Procedure

The battery of instruments was administered by advanced psychology students attending a university located in Buenos Aires city and performing their professional research practice. They were instructed to contact university students residing in Buenos Aires City and the surrounding area, who responded individually. Instruments with missing data were not included (nine protocols were eliminated). The data were analyzed with the statistical package SPSS version 17. Descriptive statistics and correlations among variables were calculated. Four clusters of complete mental well-being were obtained and the relationships among these groups and the high factors were established.

## Results

### Descriptive Statistics and Multiple Regression Analysis among Variables

Firstly, descriptive statistics of the variables included in the study were calculated (Table 2).

Secondly, successive series of stepwise multiple regression analysis were performed, including the five high factors as independent variables and the other variables of the study. The high factors peace, tenacity, cheerfulness, and erudition accounted for between 16 and 25% of the variance in well-being, depending on the type of well-being (Table 3). Regarding the psychopathological symptoms, the high factors erudition (β = −.15, *p* < .003) and peace (β = −.18, *p* < .01) accounted for 7% of the variance in the global severity index of the SCL-27 (*F*(2, 255) = 10.86, *p* < .0001). The high factors tenacity (β = .27, *p* < .0001) and erudition (β = .14, *p* < .03) accounted for 5% of academic achievement (*F*(2, 250) = 7.43, *p* < .0001), and the high factors tenacity (β = .27, *p* < .0001) and erudition (β = .19, *p* < .004) 16% of academic adjustment (*F*(2, 255) = 24.50, *p* < .0001).

**Table 3:** Summary of the Regression Analysis for High Factors Predicting Emotional, Personal, and Social Well-Being

Criterion	High Factors	*R*^2^	*F* (*df*)	*p*	Standardized β	*p*
Emotional well-being		0.184	20.22 (3, 255)	.0001		
	Tenacity				.25	<.0001
	Cheerfulness				.16	<.006
	Peace				.14	<.02
Psychological well-being		0.255	18.08 (5, 255)	.0001		
	Peace				.16	<.006
	Erudition				.16	<.01
	Tenacity				.16	<.02
	Cheerfulness				.13	<.04
Social well-being		0.164	24.92 (2, 255)	.0001		
	Peace				.35	<.0001
	Erudition				.12	<.04

### Complete Mental Well-Being: Cluster Analysis

**Figure 1:**
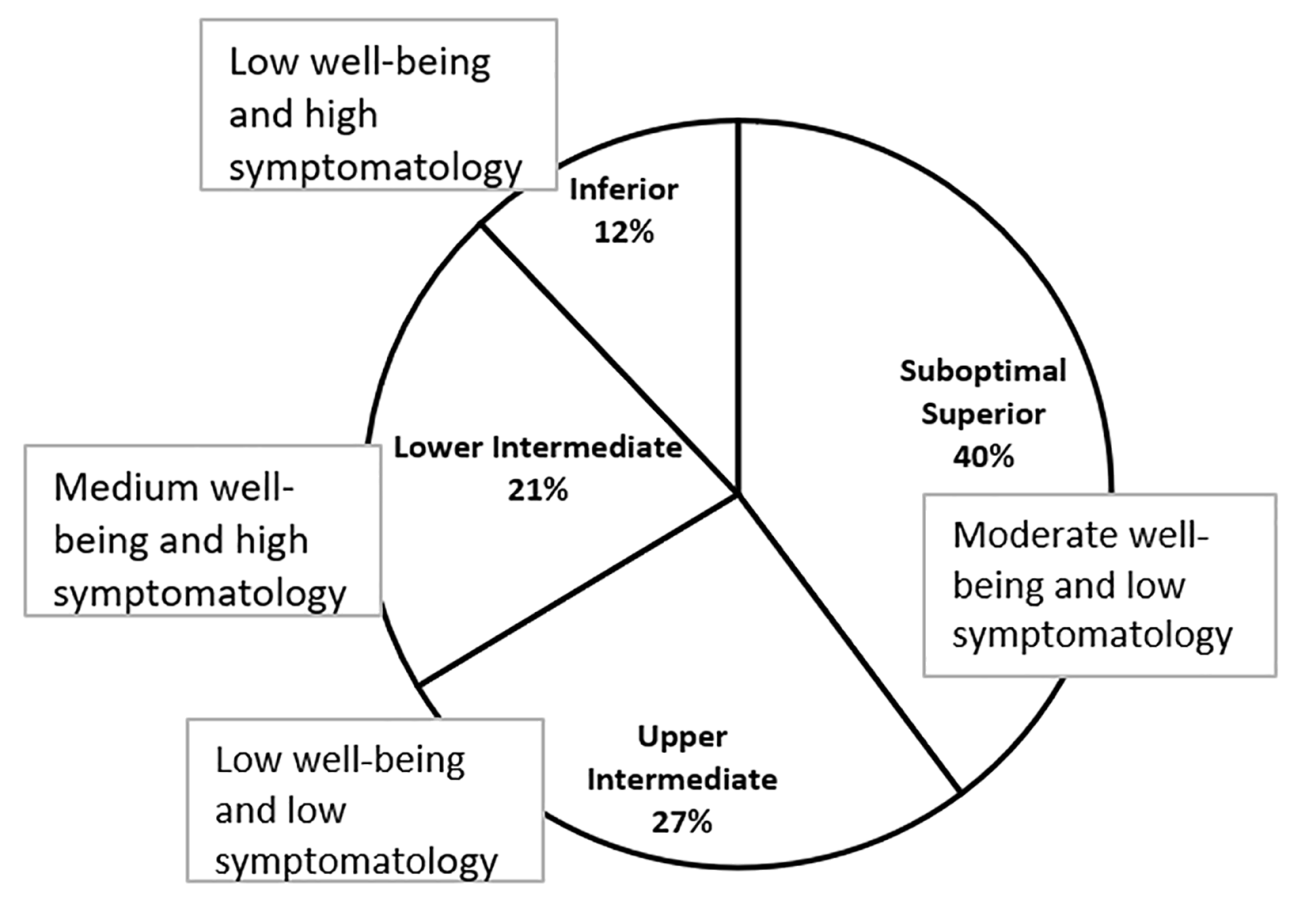
Complete mental well-being: Cluster analysis.

The next step was to obtain clusters of complete mental well-being for the complete optimal functioning, understood as the presence of high levels of both hedonic and eudemonic well-being and a low level of psychopathological symptoms. A two-step cluster analysis was performed. This exploratory analysis identifies the natural structures/grouping within a dataset and gives the optimal number of clusters as well as the quality of the fit of the groups (Pérez, 2011; Rubio-Hurtado & Vilà-Baños, 2017). The log-likelihood distance measure and the Bayesian information criterion (BIC) were used. Four clusters were identified for complete mental well-being for optimal functioning, with a goodness of fit of ≤.5. These clusters comprised superior, intermediate, and inferior levels of complete mental well-being, and, in turn, the intermediate level was subdivided into upper and lower intermediate. Figure 1 displays the clusters obtained.

The first cluster was composed of 39.8% of the cases (*n* = 102), the group with the largest number of participants. The members of this group had moderate hedonic and eudemonic well-being and a low level of psychopathological symptomatology. This cluster was named *Suboptimal Superior Level of Complete Mental Well-being* (i.e., moderate well-being and low symptomatology).

The second cluster was composed of 26.6% of the cases (*n* = 68). This group consisted of people with low levels of hedonic and eudemonic well-being and with a low level of psychopathological symptoms. This cluster was labeled *Upper Intermediate Level of Complete Mental Well-being* (i.e., low well-being and low symptomatology).

The third cluster grouped 21.5% of the cases (*n* = 55). This group reported an average level of hedonic and eudemonic well-being and above average level of psychopathological symptomatology. This cluster was in some way similar to the upper-intermediate level mentioned above and was named *Lower Intermediate Level of Complete Mental Well-being* (i.e., medium well-being and high symptomatology).

The fourth cluster consisted of 12.1% of the participants (*n* = 31). This group had very low levels of hedonic and eudemonic well-being and very high levels of psychopathological symptomatology. This cluster grouped individuals with the worst psychological profile and was labeled *Inferior Level of Complete Mental Well-being* (i.e., low well-being and high psychopathological symptomatology). In general, this cluster analysis showed that the participants could be placed along a continuum of complete mental health, from the positive pole of complete mental health to the negative pole of complete mental health. The positive pole is characterized by the presence of high hedonic and eudemonic well-being and low psychopathological symptomatology, while the negative pole is characterized by the presence of low hedonic and eudemonic well-being and the presence of high psychopathological symptoms.

### Relationships Among Clusters of Complete Mental Well-being and Sociodemographic Variables, High Factors, and Academic Variables

In the clusters of Complete Mental Well-being (CMW), no differences were found by gender (χ^2^(3, *N* = 256) = 0.80, *ns*) and age (*F*(3, 252) = 0.41, *ns*).

Associations between CMW groups and the HFM factors were analyzed. A MANOVA was conducted, comparing the five high factors across the CMW groups. Statistically significant differences were observed among the groups of CMW (Wilks’ Lambda = .73, *F*(5, 248) = 5.52, *p* < .001, η^2^ = .010). Univariate contrasts and post-hoc analyses were performed afterward. Significant differences were found among all clusters of CMW and the high factors. In most cases, the high factors of the HFM allowed us to differentiate between all the groups, as follows:

For the erudition factor (*F*(3, 252) = 12.61, *p* < .001, η^2^ = .13), post-hoc analyses (Tukey-b, *p* < .05) showed differences between the four groups of CMW. The highest scores on erudition were found in the group with the highest CMW, while the lowest scores on erudition were present in the group with the lowest CMW.

For cheerfulness (*F*(3, 252) = 7.58, *p* < .001, η^2^ = .08), post-hoc contrast (Tukey-b, *p* < .05) showed differences between the inferior CMW group and the other three groups. The group with the poorest CMW presented the lowest levels of the cheerfulness factor.

For honesty (*F*(3, 252) = 9.81, *p* < .001, η^2^ = .11), post-hoc analyses (Tukey-b, *p* < .05) showed differences between the groups with higher CMW for optimal functioning (superior and upper intermediate CMW) and the groups with lower CMW (lower intermediate and inferior CMW). The highest scores on the honesty factor were reported by individuals with better CMW, while the lowest scores were found among those with poorer CMW.

For tenacity (*F*(3, 252) = 12.50, *p* < .001, η^2^ = .13), the post-hoc contrast (Tukey-b, *p* < .05) showed differences among the groups with better CMW (superior and upper intermediate CMW) and those with worse levels of CMW (lower intermediate and inferior CMW). The highest scores on the tenacity factor were reported by individuals with better CMW, while the lowest levels of tenacity were found in those with poorer CMW.

For peace (*F*(3, 252) = 10.99, *p* < .001, η^2^ = .12), the post-hoc analyses (Tukey-b, *p* < .05) revealed differences in this high factor among the superior, intermediate, and inferior groups of CMW. The highest scores on the peace factor were reported by individuals with better CMW and the lowest scores by those with poorer CMW. Therefore the better the CMW, the higher the high factor.

Finally, no differences in academic achievement (*F*(3, 252) = 0.94, *ns*) but differences in perceived academic adjustment (*F*(3, 252) = 13.53, *p* < .01) were found, the greatest differences being among superior, intermediate, and inferior CMW groups (Tukey-b, *p* < .05). While the highest academic adjustment was observed in the superior CMW group, the lowest levels were found in the inferior CMW group.

## Discussion

The HFM is a model of positive personality traits with broad potential. Previous studies confirmed the replicability of the factor structure in an adult population and the incremental validity for the personality factors and facets of the Big Five model for predicting different types of well-being (Cosentino & Castro Solano, 2017). Moreover, the HFM factors showed the ability to discriminate between people with and without pathological personality traits. They have been considered mental health protective factors and have also been associated with low risks of medical illness (Castro Solano & Cosentino, 2017). Particularly, peace (the positive trait of balance) and cheerfulness (the positive trait of excitement) were the most strongly associated with the low risk of both psychological and medical illness.

The main contribution of this research was to provide additional validity to the HFM. To investigate whether the high factors, besides being protective of mental health and promoters of well-being, were able to predict academic variables, a sample of university students was studied. As expected, the results of this study showed that the high factors are not only related to well-being in an adult population but are also associated with academic adjustment to university life and academic achievement.

Tenacity (the positive trait of volition) and erudition (the positive trait of knowledge) are two positive factors of personality strongly related to the perception of adjustment to university life. Young adults with high levels of effort, dedication, and persistence, and are intelligent, educated, and resourceful show good academic skills to cope with university life and feel satisfied with their progress in college. Similarly, tenacity and erudition are positively associated with university grades.

The personality model of the Big Five posits that human personality is composed of five basic dimensions named neuroticism, extraversion, agreeableness, openness to experience, and conscientiousness (Costa & McCrae, 1992). Among these personality traits, conscientiousness is the predictor most frequently associated with both academic success and organizational success (e.g., Zimmerman, 2008). Individuals with high scores on conscientious tend to be hardworking, systematic, and dutiful (Trautwein, Lüdtke, Roberts, Schnyder, & Niggli, 2009), features that contribute to students' success. This trait is also important in student retention. A meta-analysis conducted by Trapmann, Hell, Hirn, and Schuler (2007) concluded that conscientiousness is a constant predictor of college grades. Trautwein’s et al. (2009) study suggests that conscientiousness is also positively associated with academic achievement. The tenacity factor of the HFM (expressed in positive characteristics such as dedication, persistence, effort, and industriousness) is conceptually and empirically related to the conscientiousness trait (Cosentino & Castro Solano, 2017) and therefore we considered conscientiousness as a proxy variable for the tenacity positive factor. As a result, the findings of this study for the prediction of academic success are consistent with those obtained with the Big Five model (Barrick & Mount, 1991; Judge, Heller, & Mount, 2002; Tross, Harper, Osher, & Kneidinger, 2000; Zimmerman, 2008). Along the same line, another positive trait associated with academic adjustment was found to be erudition (expressed in positive characteristics such as intelligent, wise, visionary, cultured, genius, and ingenious). The self-perception that one has positive qualities related to erudition conceptually refers to self-efficacy related to knowledge. The relationship between self-efficacy and academic performance is extensively documented in the literature (Bandura, Barbaranelli, Caprara, & Pastorelli, 1996; Chemers, Hu, & Garcia, 2001; Greene, Miller, Crowson, Duke, & Akey, 2004; Pintrich & DeGroot, 1990; Schunk, 1994; Sharma & Silbereisen, 2007; Zimmerman & Bandura, 1994).

In short, the results of this study provide additional validity to the HFM as a predictor of CMW for the complete optimal functioning (i.e., low presence of psychopathological symptoms and high hedonic and eudemonic well-being). The five positive traits adequately identified different gradations in the optimal functioning continuum (from positive complete mental health to negative complete mental health). High scores for the high factors were found in the most adaptive groups while low scores on these factors were found in the least adaptive groups, that is, those with less mental well-being. Therefore, the results of this research provide additional evidence to support previous findings that high factors are indicators not only of mental health but also of lower risks of medical illness in the general population (Castro Solano & Cosentino, 2017). In this sense, previous conclusions could be generalized to university students.

### Limitations and Future Perspectives

A possible limitation of our research is that only self-reported academic grades were used for academic achievement. Using objective grades obtained directly from educational institutions might serve as a more reliable source of information. In addition, this study was cross-sectional. A longitudinal study could have provided more conclusive evidence of the causal relationships in which the high factors could be considered independent variables. Besides, because this study aimed to show the links of the high factors with various positive variables, there was no control for the factors of the Big Five model. Further studies could include the Big Five factors as control variables to determine the contribution that the high factors make to the variables studied here, beyond the explanation of the Big Five factors. Future studies should also address the relationship between the HFM and academic dropout, as well as the behavior of high factors during a university academic period. Additionally, the role of the HFM as a predictor of good job performance could be studied.

### Concluding Remarks

To summarize, the assessment of positive traits derived from a psycho-lexical approach, evaluated through a brief measurement instrument such as the HFI, can predict both complete mental health for optimal functioning and other positive outcomes such as academic performance (e.g., academic adjustment and achievement). The items of the HFI use descriptors of the HFM model that are easy to understand and rapid to respond, taking no more than 5 minutes to fill out. Consequently, the HFI can be considered a reliable, valid, and efficient assessment of positive personality traits and a good alternative to the classic inventories and other personality assessment techniques that consist of a large number of items and/or more complex evaluation.
